# The same boat, different storm: stress volatile emissions in response to biotrophic fungal infections in primary and alternate hosts

**DOI:** 10.1080/15592324.2023.2217030

**Published:** 2023-05-26

**Authors:** Hassan Yusuf Sulaiman, Eve Runno-Paurson, Ülo Niinemets

**Affiliations:** a Chair of Crop Science and Plant Biology, Estonian University of Life Sciences, Tartu, Estonia; b Estonian Academy of Sciences, Tallinn, Estonia

**Keywords:** defense signaling pathways, host-pathogen interaction, isoprene, infection severity, limiting nutrient, pathogen stress, photosynthesis, volatile organic compounds

## Abstract

Rust infection results in stress volatile emissions, but due to the complexity of host-pathogen interaction and variations in innate defense and capacity to induce defense, biochemical responses can vary among host species. Fungal-dependent modifications in volatile emissions have been well documented in numerous host species, but how emission responses vary among host species is poorly understood. Our recent experiments demonstrated that the obligate biotrophic crown rust fungus (P. coronata) differently activated primary and secondary metabolic pathways in its primary host Avena sativa and alternate host Rhamnus frangula. In A. sativa, emissions of methyl jasmonate, short-chained lipoxygenase products, long-chained saturated fatty acid derivatives, mono- and sesquiterpenes, carotenoid breakdown products, and benzenoids were initially elicited in an infection severity-dependent manner, but the emissions decreased under severe infection and photosynthesis was almost completely inhibited. In R. frangula, infection resulted in low-level induction of stress volatile emissions, but surprisingly, in enhanced constitutive isoprene emissions, and even severely-infected leaves maintained a certain photosynthesis rate. Thus, the same pathogen elicited a much stronger response in the primary than in the alternate host. We argue that future work should focus on resolving mechanisms of different fungal tolerance and resilience among primary and secondary hosts.

## Introduction

Numerous studies have demonstrated that pathogen attacks negatively impact photosynthesis and activate different hormonal pathways including jasmonic acid (JA) and/or salicylic acid (SA) signaling and alteration of the activity of different secondary metabolic pathways^1,2^. This results in enhanced emissions of various stress marker compounds and defensive metabolites such as short-chained lipoxygenase (LOX) pathway volatiles (also called ’green leaf volatiles’)^3,4^, mono- and sesquiterpenes^5–7^, benzenoids^1,8^, and carotenoid breakdown products from the geranylgeranyl diphosphate (GGDP) pathway^3,9^.

Many widespread fungal pathogens such as *Melampsora* spp. and *Puccinia* spp. are multi-host (heterecious) pathogens requiring two phylogenetically different hosts, primary and secondary host to complete their life cycle^10–12^. Most studies looking at quantitative relationships between infection severity by multi-host pathogens and stress volatile emissions have focused on single hosts (Toome *et al*.,^13,14^. However, physiological and biochemical responses can vary among host species of multi-host pathogens at different parts of their life cycle. Such variations might result from differences in adaptive responses in different hosts, interspecific differences in host-pathogen interactions and different pathogen pressures on different hosts^15,16^. In addition, host differences in the expression of constitutive defenses and capacity to induce defense responses can result in divergent elicitation of volatile emissions in different host species^2^. Regarding volatiles, variations in the degree of constitutive isoprene emissions can give rise to differences in the induction of emissions of stress-elicited isoprenoids in different hosts^5,7,14,17^.

Phylogenetically different hosts also have different ecological requirements, implying that heterecious fungal infections can impact a range of ecosystems^18^. Furthermore, many primary hosts are widespread crops, and thus, information on fungal stress responses of different host species is important in developing rust fungus-resistant crops^19,20^. This is especially relevant given that heterecious biotrophic fungi are suggested to exert more severe stress on primary hosts than on alternate hosts, as the pathogens only transit the alternate host^21^. This evidence collectively suggests that potential differences in the physiological responses of primary hosts and alternate hosts of fungal pathogens need to be carefully scrutinized.

## *Puccinia coronata* infection as a model to study fungal-induced physiological changes in different hosts

We conducted experiments to investigate how a heterecious obligate biotrophic fungus, crown rust (*P. coronata*) modifies volatile organic compound (VOC) emission profiles at different levels of infection severity in its primary and secondary hosts^22^. Its primary host, where the asexual reproduction of the fungus takes place, is the annual grass, cultivated oat (*Avena sativa* L., Gramineae), and the alternate host, where the karyogamy and meiosis of the fungus occur, is the shrub to small tree alder buckthorn (*Rhamnus frangula* L., syn. *Frangula alnus* P. Mill., *Rhamnaceae*). We used *P. coronata* as the fungal model organism as it is highly virulent with a considerable rise in virulence reported recently^23,24^Sowa and Paczos-Grzęda, 2021). Also, the host species of *P. coronata* have varying degrees of constitutive emissions of isoprene; *A. sativa* is a weak emitter, whereas *R. frangula* is a moderately strong emitter^22^.

**Figure 1.**
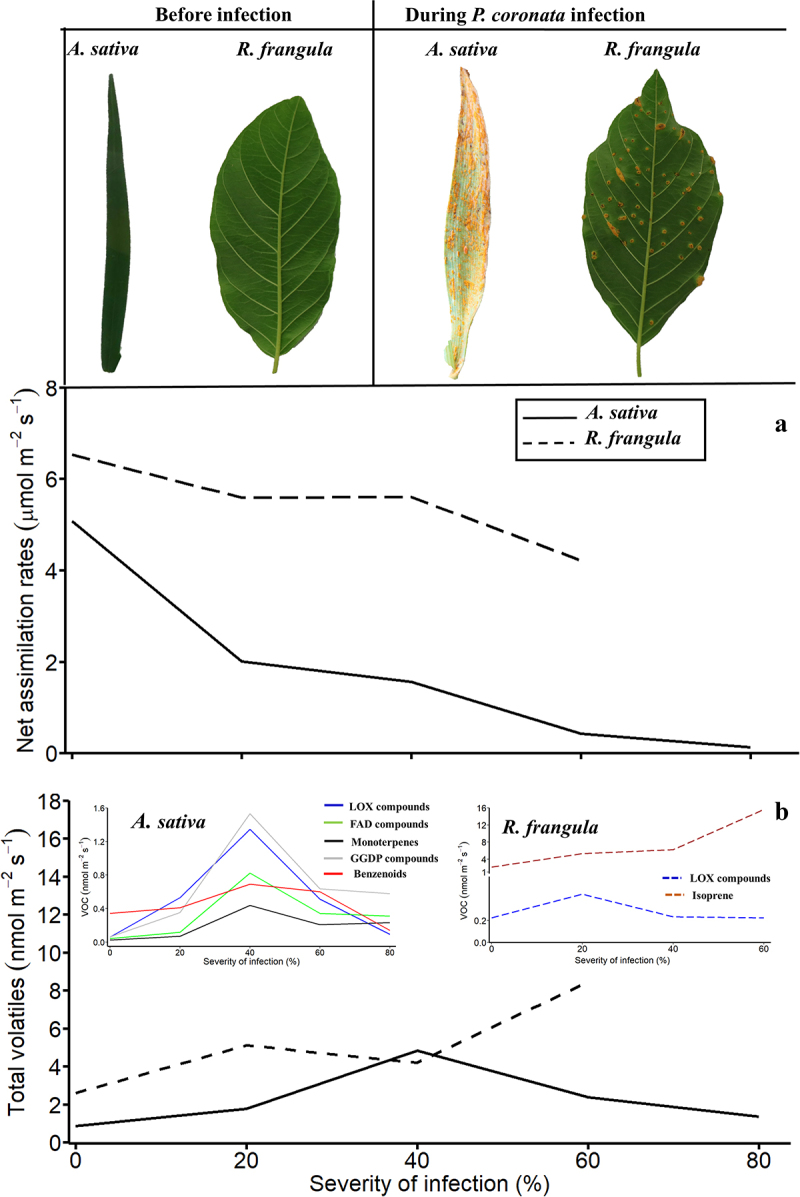
Changes in leaf light-saturated net assimilation rate (A) and total volatile emission (B) in the primary host, the annual grass Avena sativa, and the alternate host, the shrub R. frangula, under different severity of the crown rust Puccinia coronata infection. The insets in (B) show the severity-dependent emissions of different volatile groups including short-chained lipoxygenase (LOX) pathway compounds, methyl jasmonate (MeJA), long-chained saturated fatty acid-derived (FAD) compounds, monoterpenes, geranylgeranyl diphosphate pathway (GGDP) compounds and benzenoids in A. sativa and R. frangula. The severity of infection was quantified as the percentage of the total chlorotic and necrotic area of the leaf.

**Figure 2.**
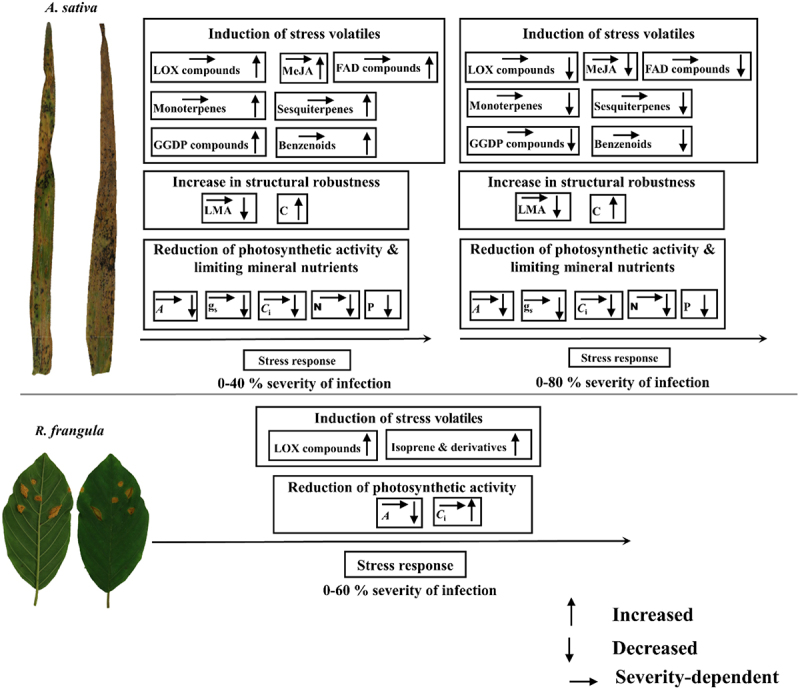
A generalized model of P. coronata infection severity-dependent responses of photosynthetic traits and stress volatile emissions in the primary host A. sativa and alternate host R. frangula. This model shows that the rate of photosynthesis (A) in the primary host is reduced due to stomatal limitations (decreases in stomatal conductance, g_s,_ and intercellular concentrations of CO_2_, C_i_). Reductions in photosynthetic activity are escalated by fungal absorption of limiting mineral nutrients and loss of photosynthetic biomass, indicated by decreases in leaf dry mass per unit area (LMA), due to fungal consumption of leaf biomass. Loss of photosynthetic function is accompanied by accumulation of carbon-rich secondary metabolites e.g. phenolics such as lignin in cell walls that enhances leaf mechanical robustness and reduces cell wall diffusion conductance for CO_2_. in the alternate host, decreases in photosynthesis are due to reductions in photosynthetic capacity. In the primary host, fungal-induced damages and hypersensitive responses trigger a burst of lipoxygenase (LOX) volatiles and the activation of defense signaling associated with jasmonic acid (JA) accumulation. This leads to the induction of emissions of stress volatiles including mono- and sesquiterpenes from chloroplastic and cytosolic terpene synthesis pathways and benzenoids from the shikimate pathway. Additionally, fungal-induced oxidative stress enhances the release of long-chained saturated fatty acid (FAD) derivatives and geranylgeranyl diphosphate (GGDP) pathway volatiles (carotenoid breakdown products). The emissions of volatiles increase with increasing severity of fungal infection, however, under severe infections, the induction of stress volatiles decreases due to substrate limitation that occurs as a result of inhibition of photosynthesis and cessations of physiological activities in necrotic leaf regions. In the resistant alternate host, due to low oxidative stress, LOX emissions are only elicited to a minor degree. Differently from the enhancement of terpene emissions in A. sativa, in R. frangula, constitutive emissions of isoprene are enhanced upon rust infection, differently from pathogen responses observed in other constitutive isoprene emitters.

We measured photosynthetic characteristics (light-saturated stomatal conductance, *g*_s,_ and net assimilation rate, *A*) and emissions of VOC simultaneously in leaves with varying severity of *P. coronata* infections using a custom-made gas exchange system designed for trace gas sampling, and identified different volatile compounds using gas-chromatography mass-spectrometry^22^. Additionally, we quantified mineral nutrients (nitrogen and phosphorous) and carbon contents per leaf dry mass, and leaf dry mass per area (LMA) in the different host species as these variables define the structural and chemical controls on photosynthesis, carbon sink and structural investment^22^. In these experiments, the severity of the infection, measured by the total leaf area covered by the classical rust symptoms, chlorosis, and necrosis (total visible damaged leaf area, *D*_A_), ranged from 0 (non-infected) to ∼80% in *A. sativa* and from 0 to ∼60% in *R. frangula* (Fig. 1 and 2 for images of representative infected leaves). In total, 15 leaves of *R. frangula* (three non-infected control and 12 infected leaves) and 23 leaves of *A. sativa* cv. ‘Kalle’ (three non-infected control and 20 infected leaves) with varying degrees of infection were measured^22^.

In *R. frangula*, *A* decreased with increasing severity of the infection and the reductions were primarily due to limitations of photosynthetic capacity (Fig. 1A and 2;^22^. In *A. sativa*, fungal-induced stomatal limitations resulted in decreases in photosynthetic activity at all levels of infections (Figures 1A and 2). However, under severe infection, *g*_s_ relative to *A* increased, indicating a certain reduction of photosynthetic capacity^22^. We observed that in *A. sativa*, but not in *R. frangula*, the reduction in photosynthetic activity was associated with decreases in rate limiting nutrients (N and P) and loss of photosynthetic biomass (Fig. 2), reflecting fungal consumption of leaf nutrient. Given that a large fraction of leaf nitrogen is invested in Rubisco, a decrease in nitrogen content typically results in a drastic reduction in photosynthetic capacity^25^. In addition, in *A. sativa*, the infection resulted in increases in the C contents of leaves (Fig. 2), suggesting the accumulation of the shikimic acid pathway-produced carbon-rich compounds such as lignin that promote defense against pathogens^26^.

## Differences in fungal activation of volatile synthesis pathways in different host species

Pathogens induce hypersensitive responses that trigger the activation of different hormonal signaling pathways, particularly SA and JA pathways that regulate local and systemic defense/stress responses^27,28^. Often, the hormonal pathway activated during pathogen infection depends on the pathogen type and its interaction with the hosts^28^. Typically, biotrophic fungi activate the SA pathway, whereas the JA pathway is activated by necrotrophic pathogens^29,30,31^. Research over the past decades has established that these pathways interact antagonistically in response to certain pathogens, in such a way that the activation of one pathway suppresses the other ^32^(Kunkel and Brooks 2022). However, recent evidence has also demonstrated synergistic interactions between SA and JA pathways in response to different pathogen attacks^33,34,35^. In particular, rust infection is associated with enhanced SA and JA accumulation due to the positive interaction of JA and SA signaling^34,35^. In this study, fungal infections induced the emissions of methyl jasmonate (MeJA) in *A. sativa* (Fig. 1B;^22^. Given that *A. sativa* emitted benzenoids (Fig. 1B), synthesis of which via the shikimate pathway is regulated by SA accumulation^2,8^, simultaneous emissions of MeJA and benzenoids reflect the synergistic activities of JA and SA pathways. In *R. frangula*, low-level MeJA emissions were constitutive^22^, suggesting constitutive expression of JA-dependent systemic responses that improve stress tolerance^27,36^.

In *A. sativa*, the induction of MeJA emission was accompanied by bursts of different LOX pathway volatiles (Figure 1B). Emissions of LOX pathway derivatives indicate cellular damage and generation of an oxidative burst^37,38,39^. In the case of *R. frangula*, emissions of LOX volatiles were only enhanced to a minor degree (Figure 1B), suggesting much lower oxidative stress. In *A. sativa*, emissions of LOX volatiles were accompanied by emissions of long-chain saturated fatty acid (FAD) derivatives (Fig. 1A), further indicating a stronger loss of membrane integrity in the primary host.

Terpenoid emissions were also differently enhanced in the primary and alternate hosts (Figures 1B and 2), further underscoring the differences in stress severity experienced by plants as well as the differential regulation of terpenoid pathway genes. In *A. sativa*, *P. coronata* enhanced the emissions of mono- and sesquiterpenes, but suppressed the emission of the oxygenated isoprene derivative methacrolein (Figures 1B and 2,^22^. In general, biotic stresses induce mono- and sesquiterpene emissions but decrease constitutive emissions of isoprene as observed in primary isoprene-emitting hosts infected by *Melampsora* spp.^13,14,40^. Surprisingly, in *R. frangula*, the impact of *P. coronata* on mono- and sesquiterpenes was minor, but the emissions of isoprene were enhanced (Figure 1B). This might indicate both the overall upregulation of the chloroplastic methyl-D-erythritol phosphate (MEP) pathway for isoprenoid synthesis or isoprene synthase activity^7,17,41,42^. Apparently, the stress threshold for elicitation of terpene synthesis was not exceeded in the alternate host, or *R. frangula* has an overall low capacity for induction of terpene emissions. Although emissions of specialized metabolites can enhance local and systemic defense responses, in some cases, low emissions of these volatiles can reflect enhanced defense^3,38,43,44^, as observed in *R. frangula* (Figures 1B and 2).

Altogether the different responsiveness of volatile formation pathways in the two hosts led to distinguished volatile fingerprints (Figures 1B and 2;^22^. In particular, in the primary host, the bouquet of volatile emissions was much richer (Figure 1B,^22^, including indicators of oxidative damage such as 2-ethyl-hexanol and (*E*)-2-hexenal, indicators of enhanced activation of terpenoid synthesis pathways^4^Kännaste *et al*.,^3^ and shikimic acid pathways such as β-pinene, *β*-farnesene, benzaldehyde, and benzothiazole^1,8^, and indicators of carotenoid breakdown such as geranyl acetone^3;9^. As other studies of volatile emission responses upon infection of heterecious fungi have looked at primary hosts, whether the observation of lower complexity of volatile profiles in infected alternate hosts is a general pattern requires further investigation.

## Scaling of volatile emissions with the severity of *P. coronata* infection

In *A. sativa*, emissions of stress volatiles increased with the severity of infection from 0 to 40% (Figures 1B and 2), suggesting stress severity-dependent elicitation of volatiles. Several previous studies have demonstrated that fungal-dependent emissions scale with the severity of infection^13,40^, implying that stress volatiles are increasingly elicited with increasing tissue damage. However, the late stages of *P. coronata* infection were characterized by expansions of necrosis, resembling hemi-biotrophy, that can lead to the inhibition of photosynthesis and overall physiological activities including volatile emissions^22,45^). We could not discriminate emissions from infected and non-infected regions of the leaf, however, it has been noted that for chronic infection, scaling of stress VOC with the severity of infection reflects emissions from damaged areas and immediate impact sites^2,5^. We observed that in severely infected *A. sativa*, photosynthesis was almost completely inhibited due to both stomatal limitation and inhibition of Rubisco activity (Figures 1A and 2). In addition to the spread of necrotic surface area, decreased photosynthesis of still functional leaf parts might have resulted in a shortage of substrates for volatile synthesis^46;22^. Correspondingly, in *A. sativa*, the elicitations of volatile emissions declined under severe infections, from 40 to ~ 80% severity of infection (Figures 1B and 2). Previously, such abolishing of volatile emissions has only been observed for necrotrophic infections^1,47,48^.

## Conclusion

It has been suggested that heterecious biotrophic fungi exert more severe stress on primary hosts than on alternate hosts, as the pathogens mainly require the alternate host for transit before infecting the primary host^21^, but the experimental evidence has been limited. We demonstrated that *P. coronata* infection impacted photosynthesis and activated biochemical pathways differently in the primary host and the alternate host. The difference in the physiological responses of the different host species demonstrates differences in the fungal stress sensitivity of the different host species. In the sensitive host, *A. sativa*, photosynthesis was almost completely inhibited under extreme infection, resulting in a major decline in the biosynthesis of volatiles^22^. The scaling of defense responses with increasing severity of infection in the primary host was characterized by an optimum, indicating that above a certain infection threshold, the defenses of the host were exhausted, resulting in escalated tissue damage and cell death.

We found a surprising increase in isoprene emissions in the infected alternate host *R. frangula*. This is different from other studies with constitutive isoprene emitters infected by heterecious fungal pathogens^5,14,40^, but in these studies, the constitutive emitters were the primary hosts. Overall, the fungal-dependent changes in photosynthetic traits and volatile emissions were greater in the primary host than in the alternate host, reflecting variations in the sensitivity of the physiological activities of the different hosts. A profound understanding of how different host species respond to heterecious biotrophic pathogens is relevant to predict fungal spread in both natural ecosystems and crops. We suggest that future assessments of the severity of infections of heterecious fungi should consider both primary and alternate hosts. Furthermore, comparisons of responses of different host species to the same pathogen can help identify promising plant molecular responses to pathogen infection as a breeding strategy for the enhancement of disease resilience in crop species and cultivars^49,50,51^.
